# Multiple pathways for SARS-CoV-2 resistance to nirmatrelvir

**DOI:** 10.1038/s41586-022-05514-2

**Published:** 2022-11-09

**Authors:** Sho Iketani, Hiroshi Mohri, Bruce Culbertson, Seo Jung Hong, Yinkai Duan, Maria I. Luck, Medini K. Annavajhala, Yicheng Guo, Zizhang Sheng, Anne-Catrin Uhlemann, Stephen P. Goff, Yosef Sabo, Haitao Yang, Alejandro Chavez, David D. Ho

**Affiliations:** 1 Aaron Diamond AIDS Research Center, Columbia University Vagelos College of Physicians and Surgeons, New York, NY USA; 2 Division of Infectious Diseases, Department of Medicine, Columbia University Vagelos College of Physicians and Surgeons, New York, NY USA; 3 Integrated Program in Cellular, Molecular, and Biomedical Studies, Columbia University Vagelos College of Physicians and Surgeons, New York, NY USA; 4 Medical Scientist Training Program, Columbia University Vagelos College of Physicians and Surgeons, New York, NY USA; 5 Department of Pathology and Cell Biology, Columbia University Vagelos College of Physicians and Surgeons, New York, NY USA; 6 Shanghai Institute for Advanced Immunochemical Studies and School of Life Science and Technology, ShanghaiTech University, Shanghai, China; 7 Department of Microbiology and Immunology, Columbia University Vagelos College of Physicians and Surgeons, New York, NY USA; 8 Department of Biochemistry and Molecular Biophysics, Columbia University Vagelos College of Physicians and Surgeons, New York, NY USA

**Keywords:** SARS-CoV-2, Antimicrobial resistance

## Abstract

Nirmatrelvir, an oral antiviral targeting the 3CL protease of SARS-CoV-2, has been demonstrated to be clinically useful against COVID-19 (refs. ^1,2^). However, because SARS-CoV-2 has evolved to become resistant to other therapeutic modalities^3–9^, there is a concern that the same could occur for nirmatrelvir. Here we examined this possibility by in vitro passaging of SARS-CoV-2 in nirmatrelvir using two independent approaches, including one on a large scale. Indeed, highly resistant viruses emerged from both and their sequences showed a multitude of 3CL protease mutations. In the experiment peformed with many replicates, 53 independent viral lineages were selected with mutations observed at 23 different residues of the enzyme. Nevertheless, several common mutational pathways to nirmatrelvir resistance were preferred, with a majority of the viruses descending from T21I, P252L or T304I as precursor mutations. Construction and analysis of 13 recombinant SARS-CoV-2 clones showed that these mutations mediated only low-level resistance, whereas greater resistance required accumulation of additional mutations. E166V mutation conferred the strongest resistance (around 100-fold), but this mutation resulted in a loss of viral replicative fitness that was restored by compensatory changes such as L50F and T21I. Our findings indicate that SARS-CoV-2 resistance to nirmatrelvir does readily arise via multiple pathways in vitro, and the specific mutations observed herein form a strong foundation from which to study the mechanism of resistance in detail and to inform the design of next-generation protease inhibitors.

## Abstract (web summary)

Nirmatrelvir, an oral antiviral targeting the 3CL protease of SARS-CoV-2, has been demonstrated to be clinically useful against COVID-19, but viral resistance to the drug was found to arise readily via multiple pathways in vitro.

## Main

The COVID-19 (coronavirus disease, 2019) pandemic has continued to affect the global populace. The rapid development and deployment of effective vaccines, as well as monoclonal antibody therapeutics beginning in late 2020, have helped greatly to curtail its impacts^10–16^. Nevertheless, the aetiologic agent, SARS-CoV-2 (severe acute respiratory syndrome coronavirus 2), has continuously evolved to develop resistance to antibody-mediated neutralization^4–8^. In particular, several recent Omicron subvariants exhibit such strong antibody resistance that vaccines have had their protection against infection dampened and a majority of current monoclonal therapeutics have lost efficacy^4,5,8^, as manifested by increasing levels of breakthrough infections in convalescing and/or vaccinated individuals^3^.

Fortunately, treatment options remain. In the United States, three antivirals have received emergency use authorization for COVID-19 treatment: remdesivir^17,18^, molnupiravir^19–21^ and nirmatrelvir^1,2^ (also known as PF-07321332, used in combination with ritonavir and marketed as PAXLOVID). The first two target the RNA-dependent RNA polymerase (RdRp) and the latter targets the 3CL protease (3CL^pro^, also known as main protease (M^pro^) and nonstructural protein 5 (nsp5)). Both enzymes are essential for the viral life cycle and are relatively conserved among coronaviruses^22,23^. Remdesivir is administered intravenously and has a reported relative risk reduction of 87% (ref. ^18^), whereas molnupiravir and nirmatrelvir are administered orally and have reported clinical efficacies of 31% (ref. ^20^) and 89% (ref. ^1^), respectively, in lowering rates of hospitalization or death. As the use of these antivirals increases there is a concern that drug resistance may arise, particularly if given as monotherapies. For remdesivir, in vitro and in vivo studies have shown mutations associated with resistance^9,24,25^, and resistance to molnupiravir or nirmatrelvir is now under active investigation. Here we report that there are multiple routes by which SARS-CoV-2 can gain resistance to nirmatrelvir in vitro.

## Nirmatrelvir resistance in Vero E6

**Fig. 1:**
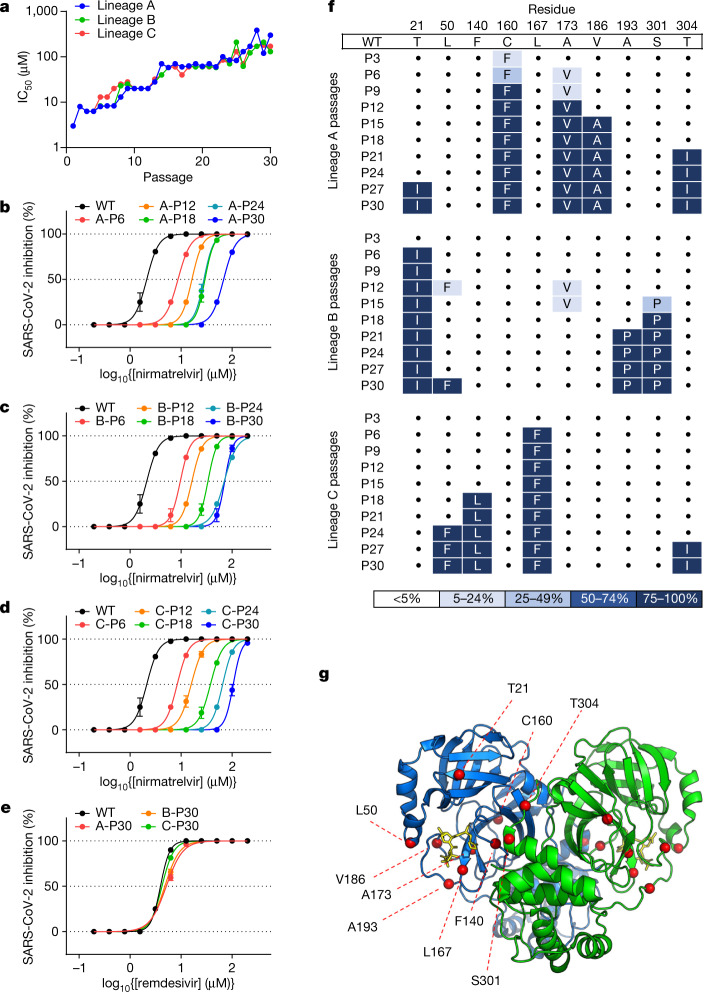
Identification of nirmatrelvir resistance in Vero E6 cells. **a**, Changes in IC_50_ during passaging of SARS-CoV-2 with nirmatrelvir. Vero E6 cells were infected in triplicate with SARS-CoV-2 (USA-WA1/2020) and passaged to fresh cells every 3 days for 30 passages (Methods). **b**–**d**, Validation of nirmatrelvir resistance for the indicated passage from each of the three lineages (A (**b**), B (**c**) and C (**d**)). **e**, Inhibition of passage 30 viruses from each lineage by remdesivir. **f**, Mutations in 3CL^pro^ found in the indicated passages from each lineage. Dots indicate WT at that residue. Mutations are shaded according to frequency. **g**, Residues mutated with passaging in Vero E6 cells overlaid onto the 3CL^pro^ structure with nirmatrelvir bound. The Cα of each mutated residue is denoted by a red sphere. The 3CL^pro^–nirmatrelvir complex was downloaded from PDB under accession code 7VH8. **a**–**e**, Error bars denote mean ± s.e.m of four technical replicates.

**Extended Data Fig. 1:**
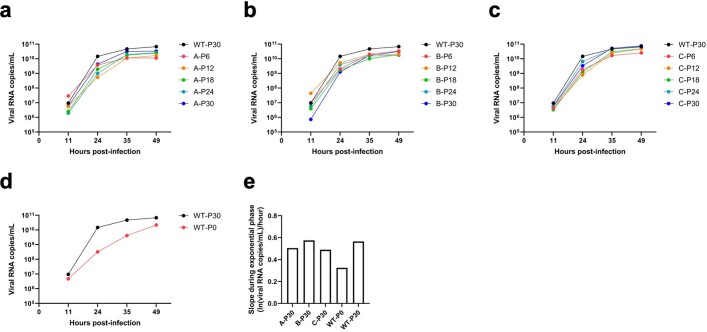
Growth assays with SARS-CoV-2 passaged in Vero E6 cells. **a**-**d**, Growth was quantified for lineage A (**a**), lineage B (**b**), lineage C (**c**), and unpassaged SARS-CoV-2 (**d**, denoted as WT-P0) in comparison to SARS-CoV-2 passaged without nirmatrelvir for 30 passages (denoted as WT-P30). Vero E6 cells were infected with 200 TCID_50_ of the indicated viruses and viral RNA was quantified at the indicated time points. **e**, The slope during the exponential phase (between 11 and 24 h post-infection) of growth for the indicated viruses.

To select for resistance to nirmatrelvir, SARS-CoV-2 (USA-WA1/2020 strain) was passaged in the presence of increasing concentrations of the drug (Methods). We conducted this initial experiment in triplicate, using Vero E6 cells because they have been one of the standard cell lines used in COVID-19 research. After 30 passages each of the three lineages demonstrated a high level of resistance, with half-maximal inhibitory concentration (IC_50_) values increasing 33- to 50-fold relative to that of the original virus (Fig. 1a–d). Examination of earlier viral passages confirmed a stepwise increase in nirmatrelvir resistance with successive passaging (Fig. 1b–d), with no evidence of resistance to remdesivir (Fig. 1e). The resistant viruses selected by passaging maintained their replicative fitness in vitro, with growth kinetics similar to those passaged without nirmatrelvir (Extended Data Fig. 1).

We then sequenced the 3CL^pro^ gene from the three viral lineages collected every three passages to investigate which mutations might confer resistance (Fig. 1f). We found that the three lineages harboured unique mutations with only one mutation, at most, overlapping between the different lineages (T21I in lineages A and B, L50F in lineages B and C and T304I in lineages A and C). The observed mutations occurred in a stepwise manner, mirroring the increases in drug resistance (Fig. 1f) and a number of them, but not all, were situated near the nirmatrelvir-binding site (Fig. 1g). Specifically, F140L and L167F were within 5 Å of nirmatrelvir. These results suggested that SARS-CoV-2 could readily develop nirmatrelvir resistance using any one of several mutational pathways.

## Nirmatrelvir resistance in Huh7-ACE2 cells

**Fig. 2:**
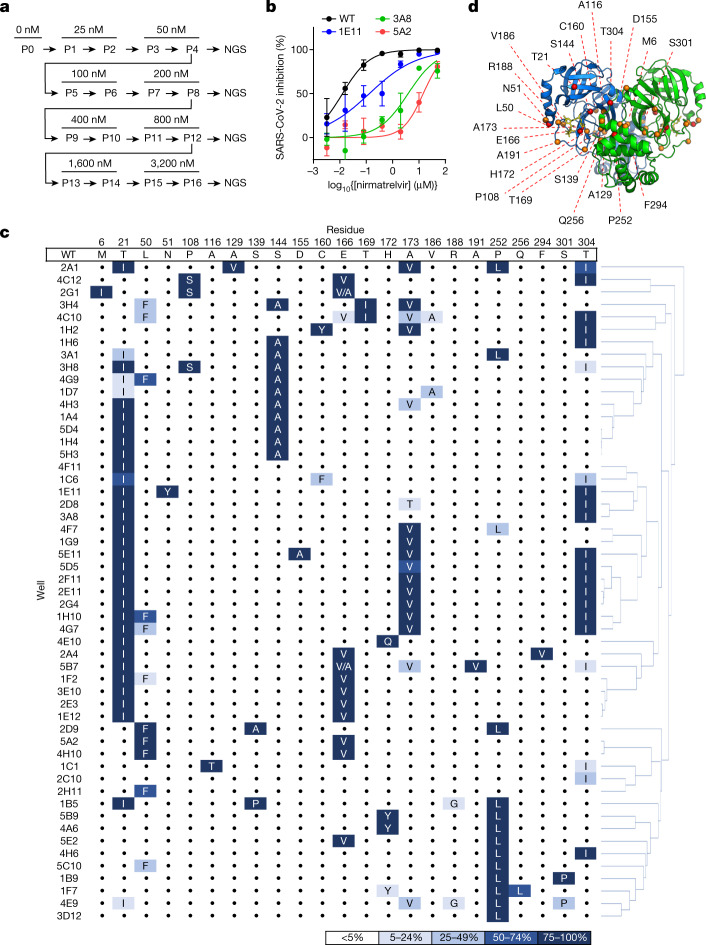
Identification of nirmatrelvir resistance at scale in Huh7-ACE2 cells. **a**, Passaging scheme: 480 wells were infected with SARS-CoV-2-mNeonGreen and passaged to fresh Huh7-ACE2 cells every 3–4 days, with the concentration of drug doubled every two passages. **b**, Validation of nirmatrelvir resistance of three wells from passage 16. These viral populations had the following mutations: 3A8 (T21I, T304I), 1E11 (T21I, N51Y, T304I) and 5A2 (L50F, E166V). See Supplementary Table 1 for exact frequencies. Representative curves from a single experiment from two biologically independent experiments are shown. Error bars denote mean ± s.e.m of three technical replicates. **c**, Mutations in 3CL^pro^ found in passage 16 from 53 wells. Dots indicate WT at that residue. Mutations are shaded according to frequency. **d**, Residues mutated in passaging in Huh7-ACE2 cells overlaid onto the 3CL^pro^ structure with nirmatrelvir bound. All 23 mutated residues across all resistant populations are indicated for any individual isolate having between one and six mutations. The Cα of each residue that was mutated is denoted by a red sphere for mutations observed more than ten times, and is denoted by an orange sphere for mutations observed fewer than ten times. The 3CL^pro^–nirmatrelvir complex was downloaded from PDB under accession code 7VH8.

**Extended Data Fig. 2:**
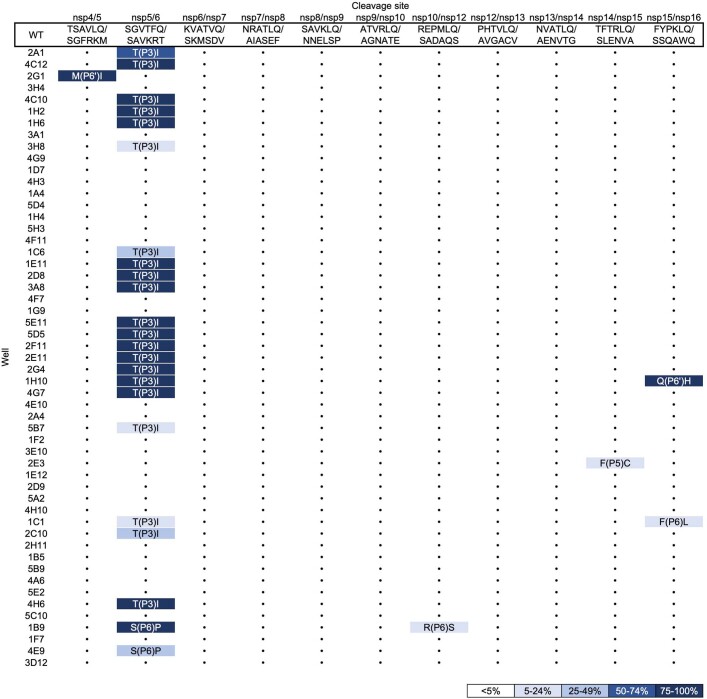
Mutations in the 11 3CL^pro^ cut sites found in passage 16 from the 53 wells passaged in Huh7-ACE2 cells. Dots indicate wild-type at that cut site. Note that nsp4/5 M(P6’)I = M6I, nsp5/6 S(P6)P = S301P, and nsp5/6 T(P3)I = T304I in 3CL^pro^.

We therefore set out to conduct another passaging experiment to select for nirmatrelvir resistance, but this time at a larger scale with many replicates to better capture the multitude of solutions that SARS-CoV-2 could adopt under drug pressure. For these later studies, we utilized Huh7-ACE2 cells to examine whether differences would arise in human cells, and because Vero E6 cells express high levels of P-glycoprotein, an efflux transporter that limits the intracellular accumulation of nirmatrelvir^26^. We passaged SARS-CoV-2-mNeonGreen (USA-WA1/2020 background with ORF7 replaced by mNeonGreen^27^) independently in 480 wells for 16 passages with increasing concentrations of nirmatrelvir over time, and viruses from every fourth passage were subjected to next-generation sequencing (NGS) (Fig. 2a and Methods). After 16 passages, varying degrees of nirmatrelvir resistance were observed as exemplified by the three viruses shown in Fig. 2b. Sequencing of 3CL^pro^ in all wells that retained mNeonGreen signal identified 53 mutant populations (Fig. 2c). Across all of these populations, mutations were observed at 23 residues within the enzyme (between one and six mutations in each isolate), both proximal (at least 5 Å; S144A, E166(A/V), H172(Q/Y) and R188G) and distal (over 5 Å) to nirmatrelvir (Fig. 2d). Whereas there was widespread diversity among the passaged populations, seven mutations appeared ten or more times across replicates: T21I, L50F, S144A, E166V, A173V, P252L and T304I. The only 3CL^pro^ cleavage site mutation frequently observed was T304I, which corresponds to the cleavage site nsp5/6 T(P3)I. Other sites were only rarely observed to mutate, suggesting that substrate cleavage site alterations are largely not responsible for nirmatrelvir resistance (Extended Data Fig. 2), with the possible exception of *cis*-cleavage.

**Fig. 3:**
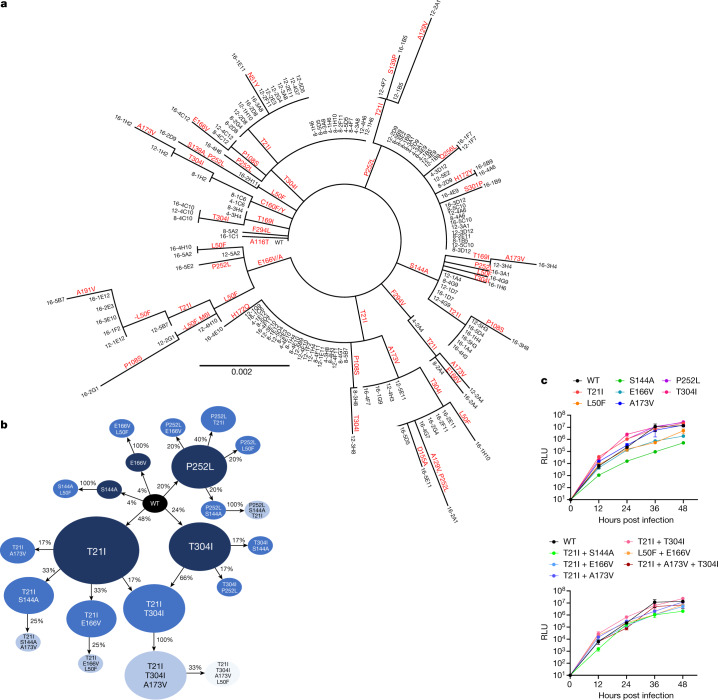
Pathways for SARS-CoV-2 resistance to nirmatrelvir. **a**, Phylogenetic tree of sequences from passaging in Huh7-ACE2 cells. Only sequences with mutations are shown. Sequences are denoted as passage number, followed by the well number. Mutations that arose along particular branches are annotated in red; ‘-’ denotes when a mutation appears to have been lost from a particular branch. **b**, Observed pathways for nirmatrelvir resistance in Huh7-ACE2 cells. The most commonly observed mutations in passage 16 were used to build these pathways (Methods and Supplementary Table 2). Nodes are shaded from dark to light, with founder mutations darker. Percentages indicate the frequency by which child nodes derive from the immediate parental node. Descendent arrows that do not sum to 100% indicate that a proportion did not advance beyond the indicated mutations in the experiment. **c**, Growth assay with recombinant live SARS-CoV-2 carrying single (top) and combination 3CL^pro^ mutations (bottom). Huh7-ACE2 cells were infected with 0.01 multiplicity of infection (MOI) of virus, and luminescence was quantified at the indicated time points. S144A, E166V and T21I + S144A are statistically significant from WT at 48 h (two-way analysis of variance with Geisser–Greenhouse correction followed by Dunnett’s multiple comparisons test; *P* = 0.0039, *P* = 0.0006, *P* = 0.0006, respectively). Representative curves from a single experiment from two biologically independent experiments are shown. Error bars denote mean ± s.e.m of three technical replicates. RLU, relative luminescence units.

Sequencing of the same wells at earlier passages showed less diversity in 3CL^pro^, with totals of 11, 16 and 22 unique mutations detected across all populations from passages 4, 8 and 12, respectively (Supplementary Table 1). Because a standard phylogenetic analysis showed a rather complex stepwise order of acquisition of mutations for each passaged lineage (Fig. 3a), we more carefully analysed the order in which mutations arose across the various lineages (Methods and Supplementary Table 1) and generated a pathway network delineating the most common routes taken by SARS-CoV-2 in vitro to develop nirmatrelvir resistance (Fig. 3b and Supplementary Table 2). The majority of these viral lineages descended initially from T21I, P252L and T304I, suggesting that these mutations may serve as ‘founder’ or ‘precursor’ mutations when drug concentrations are relatively low. Additional mutations then occurred, probably to increase the level of resistance as drug concentrations were increased and/or to compensate for reduced viral fitness. These findings indicated that, although there are multiple means by which SARS-CoV-2 can resist nirmatrelvir, several common mutational pathways are favoured.

## Nirmatrelvir resistance mutations

**Fig. 4:**
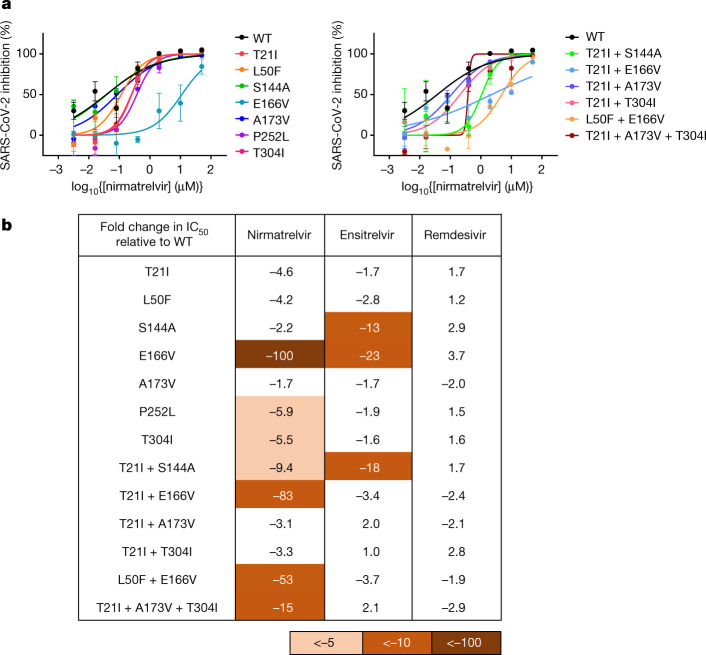
Validation of identified mutations in isogenic recombinant SARS-CoV-2. **a**, Individual inhibition curves of recombinant live SARS-CoV-2 carrying single (left) and combination 3CL^pro^ mutations (right) by nirmatrelvir. Representative curves from a single experiment from three biologically independent experiments are shown. Error bars denote mean ± s.e.m of three technical replicates. **b**, Inhibition of recombinant live SARS-CoV-2 carrying single and combination 3CL^pro^ mutations by nirmatrelvir, ensitrelvir and remdesivir. Values shown are fold change of mean values in IC_50_ relative to inhibition of WT from three biologically independent experiments.

**Extended Data Fig. 3:**
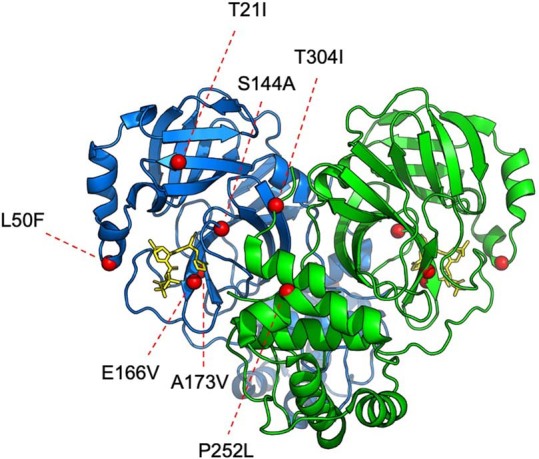
Mutations studied as isogenic recombinant SARS-CoV-2 overlaid onto the 3CL protease structure. The Cα of each residue that was mutated is denoted with a red sphere. The 3CL^pro^-nirmatrelvir complex was downloaded from PDB under accession code 7VH8.

**Extended Data Fig. 4:**
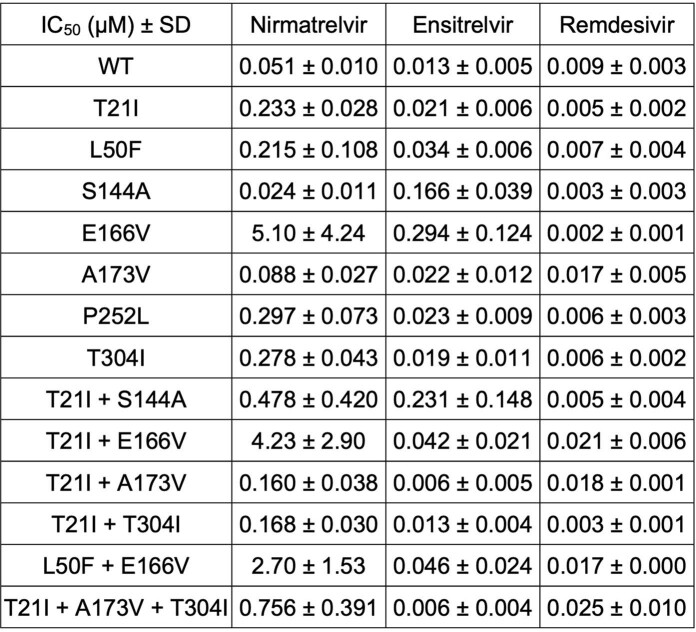
Raw IC_50_ values for recombinant live SARS-CoV-2 carrying single and combination 3CL^pro^ mutations by nirmatrelvir, ensitrelvir, and remdesivir. Mean ± SD of three biologically independent experiments are shown.

To further investigate which mutations were responsible for nirmatrelvir resistance we proceeded to generate recombinant SARS-CoV-2 clones, each containing a unique mutation or a combination of mutations. To construct the 15 mutant viruses from the first passage experiment (Fig. 1f) and the 22 mutant viruses from the second (Fig. 3a,b) would be beyond the scope of the current study. We therefore decided to focus on the seven most common single-point mutants from the large passaging study, as well as on five double mutants and one triple mutant (Extended Data Fig. 3). All viruses grew similarly to wild type (WT) in the absence of drug except for S144A, E166V and T21I + S144A, which were significantly impaired in their growth kinetics (Fig. 3c). However, both T21I + E166V and L50F + E166V replicated well with kinetics similar to WT, suggesting that T21I and L50F each compensated for the fitness loss of E166V. Of the individual mutants tested against nirmatrelvir, E166V was most resistant (100-fold) with P252L and T304I having low-level resistance (around sixfold) and S144A and A173V only minimal resistance (about threefold or less) (Fig. 4a,b and Extended Data Fig. 4). Combination of either T21I or L50F with E166V resulted in a virus that was substantially resistant to nirmatrelvir (83- and 53-fold, respectively), but with WT replicative kinetics (Fig. 3c).

**Extended Data Fig. 5:**
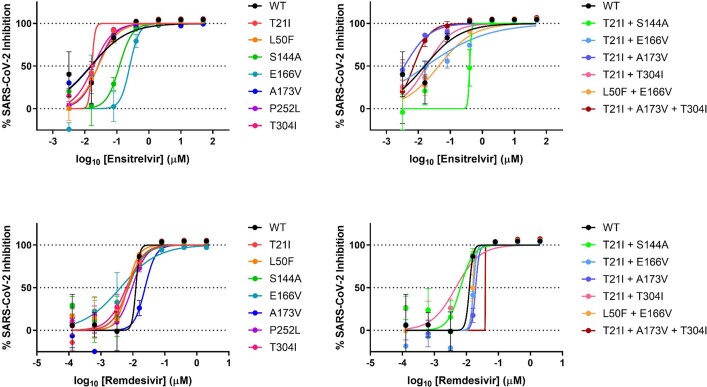
Individual inhibition curves of recombinant live SARS-CoV-2 carrying single and combination 3CL^pro^ mutations by ensitrelvir and remdesivir. Representative curves from a single experiment from three biologically independent experiments are shown. Error bars denote mean ± s.e.m of three technical replicates.

**Extended Data Fig. 6:**
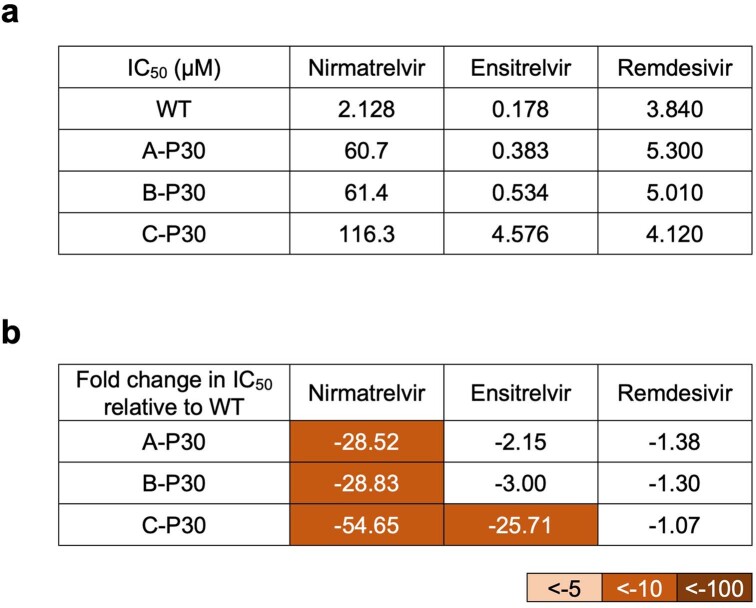
Inhibition of passage 30 of SARS-CoV-2 passaged in Vero E6 cells by nirmatrelvir, ensitrelvir, and remdesivir. **a**, Raw IC_50_ values. **b**, Fold change relative to inhibition of wild-type.

**Extended Data Fig. 7:**
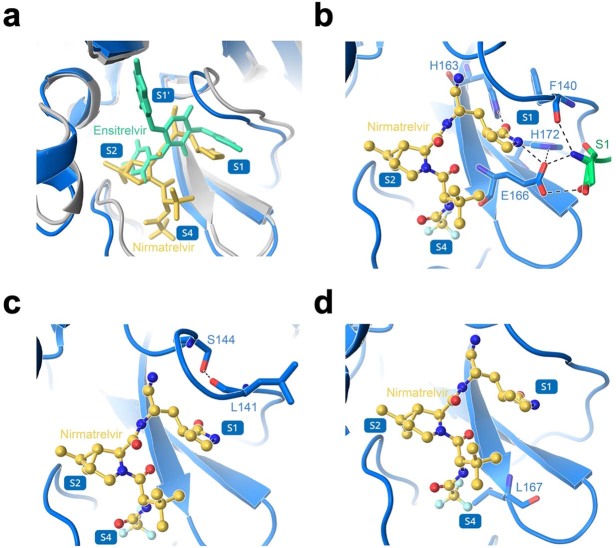
Structural analyses of 3CL^pro^ mutations. **a**, Overlay of nirmatrelvir and ensitrelvir binding to 3CL^pro^. **b**, Several of the residues involved in direct interaction with nirmatrelvir. **c**, Several of the residues involved in formation of the S1 subsite. **d**, Interaction of L167 with nirmatrelvir. In **a**-**d**, nirmatrelvir is shown in yellow, enstirelvir is shown in lime green, the 3CL^pro^-nirmatrelvir complex is shown in marine, and the 3CL^pro^-ensitrelvir complex is shown in gray. Protomer A is shown in marine and protomer B is shown in green. Hydrogen bonds are indicated as black dashes. The 3CL^pro^-nirmatrelvir complex and 3CL^pro^-ensitrelvir complex were downloaded from PDB under accession codes 7VH8 and 7VU6, respectively.

We next tested this panel of viruses against ensitrelvir^28^ (also known as S-217622), another 3CL protease inhibitor that has demonstrated clinical efficacy^29^, for cross-resistance, together with remdesivir as control. Only S144A, E166V and T21I + S144A showed substantial (13- to 23-fold) cross-resistance to ensitrelvir (Fig. 4b and Extended Data Figs. 4 and 5). As expected, none of these mutations conferred resistance to remdesivir. We additionally tested the passage 30 viruses resulting from the initial selection experiment in Vero E6 cells (Fig. 1) against these two inhibitors. Again, all three lineages were as susceptible to remdesivir as WT and only lineage C (L50F + F140L + L167F + T304I) showed cross-resistance to ensitrelvir (approximately 25-fold) (Extended Data Fig. 6). This may be due to F140L, because L50F and T304I did not demonstrate ensitrelvir resistance (Fig. 4b) and L167 does not contact ensitrelvir (see below). Together these results suggest that some mutations, such as E166V, can confer a high degree of nirmatrelvir resistance alone whereas others, such as T21I, P252L and T304I, confer only low levels of resistance individually. The degree of cross-resistance to ensitrelvir was variable among the tested mutant viruses, probably due to the differences in binding of these drugs to the substrate-binding site of 3CL^pro^ (Extended Data Fig. 7a). Nevertheless, it is clear that selection for nirmatrelvir resistance can yield mutations that confer cross-resistance to other inhibitors of clinical interest as well.

To begin to understand the mechanisms underlying the resistance conferred by these mutations, we considered their structural context. Nirmatrelvir and ensitrelvir both bind within the substrate-binding site, but in differing modes, which may have resulted in the differences observed in the inhibition profiles of the mutants (Fig. 4b and Extended Data Fig. 7a). E166 directly interacts with the lactam ring of nirmatrelvir via hydrogen bonding, and the valine substitution at this position may abrogate some of these interactions to result in the strong drug resistance observed (Extended Data Fig. 7b). E166 is also able to form hydrogen bonds with the first residue (S1) of the neighbouring protomer and is therefore involved in dimerization, which is essential for protease activity because 3CL^pro^ functions as a homodimer^30^. The disruption of hydrogen-bonding interactions (Extended Data Fig. 7b) may explain the reduced fitness of the E166V mutant (Fig. 3c). The side chain of S144 forms a hydrogen bond with the main chain of L141 to stabilize the S1 subsite of the substrate-binding site, so the S144A mutation may disorder this region and hamper the binding of both nirmatrelvir and ensitrelvir (Extended Data Fig. 7c), although it is not clear why this requires the T21I mutation in conjunction. L167 participates in the formation of the S4 subsite, and the L167F mutation may cause a steric clash with nirmatrelvir (Extended Data Fig. 7d). However, because ensitrelvir does not extend into the S4 subsite, this mutation may not be responsible for the cross-resistance observed in lineage C (Extended Data Fig. 6). Because F140 interacts by π-π stacking interactions with H163, which directly interacts with both nirmatrelvir and ensitrelvir, the F140L mutation may abrogate this interaction, resulting in resistance (Extended Data Figs. 6 and 7a,b). For a number of these mutations, however, it is not immediately apparent how they confer drug resistance given that they are distant from the substrate-binding site where the drugs bind (Extended Data Fig. 3).

**Extended Data Fig. 8:**
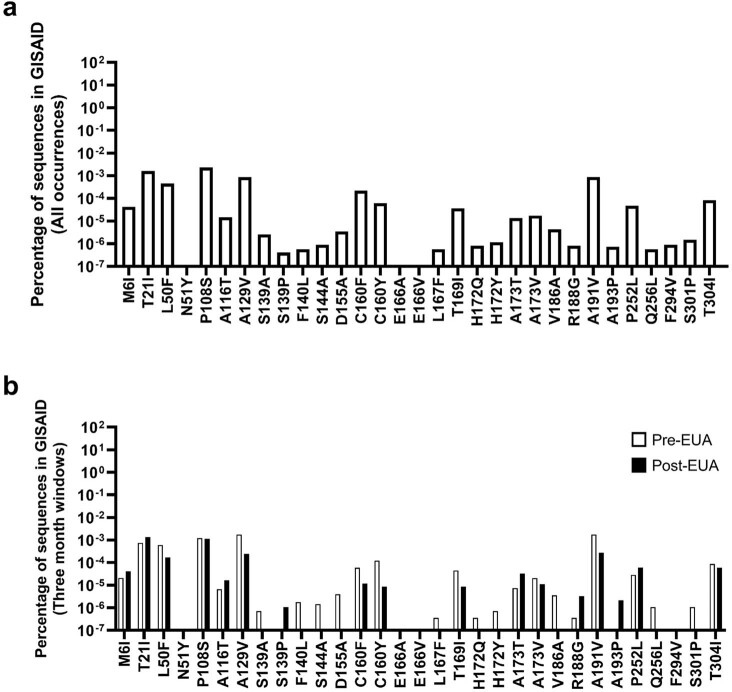
Frequencies of identified 3CL^pro^ mutations in GISAID. **a**, All occurrences of the indicated mutations were tabulated from GISAID. **b**, All occurrences of the indicated mutations were tabulated from GISAID in the three months prior to EUA (9/22/2021 to 12/22/2021) or after EUA (3/26/2022 to 6/26/2022).

Finally, we compared the mutations identified in this study with clinical SARS-CoV-2 sequences reported to the Global Initiative on Sharing Avian Influenza Data^31^. Nearly all of the mutations we have identified were observed among viruses circulating in the population, albeit at low frequencies (Extended Data Fig. 8a). Comparing the frequencies of these mutations in periods before and after authorization of the combination of nirmatrelvir and ritonavir (PAXLOVID) did not show an appreciable increase in the observed mutations (Extended Data Fig. 8b).

## Discussion

Because antibody-based interventions for SARS-CoV-2 face increasing resistance from the emergence of variants of concern, antivirals with alternative modes of action have increased in importance. Nirmatrelvir, as an oral antiviral targeting 3CL^pro^, is a therapeutic that has shown high efficacy in lowering severe disease and hospitalization in infected persons who are at high risk and not vaccinated^1,2^. Indeed, it is the antiviral drug most commonly used to treat COVID-19 today^32^. Given the adaptations that the virus has already exhibited to other modes of treatment^3–9^, it is clinically important to understand the mechanisms by which nirmatrelvir resistance can occur. The results presented herein demonstrate that in vitro high-level resistance to nirmatrelvir can readily be achieved by SARS-CoV-2, and that this can occur in a multitude of ways. This finding is consistent with our previous report on the extensive plasticity of 3CL^pro^, as discovered by deep mutational scanning^33^.

In both Vero E6 cells (Fig. 1) and Huh7-ACE2 cells (Fig. 2), multiple lineages with nonoverlapping mutations evolved under increasing drug pressure, consistent with that seen in similar small-scale studies^24,25,34,35^. Conducting selection at scale, however, showed that there are multiple mutational pathways to nirmatrelvir resistance but with several common trajectories preferred (Figs. 2c and 3a,b). A majority of lineages descended from viruses that acquired T21I, P252L or T304I as an initial mutation. Recombinant SARS-CoV-2, constructed to contain each of these point mutants, exhibited low-level resistance (Fig. 4a,b), suggesting that each of these precursor mutations may have allowed the virus to tolerate low concentrations of nirmatrelvir but required additional mutations as drug pressure was increased. Notably, all three of these mutations are somewhat distal (over 5 Å) from nirmatrelvir (Fig. 2d) and their mechanism for resistance is not evident without additional studies. We note, however, that T304 corresponds to the P3 site on the nsp5/6 cleavage substrate for 3CL^pro^ of both SARS-CoV and SARS-CoV-2 (Extended Data Fig. 2). Although the P3 site is exposed to solvent and is thus not considered to confer stringent substrate specificity, it has been shown that a suitable functional group (such as the side chain of isoleucine) at the P3 site can assist in increasing inhibitor/substrate potency and selectivity for 3CL^pro^ (refs. ^36–38^). Therefore, it is possible that T304I could facilitate binding of the nsp5/6 cleavage site or promote the autocleavage process. The differing mutations observed between the two cell lines further emphasize the complexity and variety of pathways leading to nirmatrelvir resistance, although it is not yet clear why certain mutations are specific to the Vero E6 cell line.

Analyses with isogenic mutants also showed that several mutations are responsible for the observed nirmatrelvir resistance, with the E166V mutation conferring the most resistance (100-fold) (Fig. 4b), as reported elsewhere^33,35^. This mutation also conferred a degree of cross-resistance to ensitrelvir, another clinically relevant 3CL^pro^ inhibitor^28,29^. The mechanism of resistance of E166V can be explained because it resides in the substrate-binding site, and valine substitution disrupts its hydrogen bonding to the lactam ring of nirmatrelvir (Extended Data Fig. 7b). However, this mutation lowered the replicative fitness of the virus in vitro (Fig. 3c), perhaps because of a loss of interaction with the first residue of the neighbouring protomer in dimerization (Extended Data Fig. 7b)^30^. Importantly, replicative fitness was restored when T21I or L50F was added (Fig. 3c), with no significant impact on drug resistance (Fig. 4b). How these two mutations compensate for the fitness loss of E166V remains unknown. It is worth mentioning that the E166V mutation was reportedly found in viral isolates from several PAXLOVID-treated individuals in the EPIC-HR clinical trial^1^ (see Fact Sheet for Healthcare Providers: Emergency Use Authorization for PAXLOVID, revised 6 July 2022 (ref. ^39^)).

We have also found that a number of additional mutations could confer resistance to nirmatrelvir in vitro. T21I + S144A mediated not only significant resistance to nirmatrelvir but also cross-resistance to ensitrelvir (Fig. 4b), but this virus exhibited slower growth kinetics (Fig. 3c). Likewise, we inferred that both L167F and F140L were probably mediating drug resistance in the C-P30 lineage of the first in vitro passaging experiment (Fig. 1f) as discussed above, along with possible structural explanations. It is clear, nevertheless, that we have studied only a limited number of the mutational pathways taken by SARS-CoV-2 to evade nirmatrelvir. Furthermore, many of the mutations shown by our study are without a straightforward structural explanation at this time, and indeed, whereas other in vitro or in silico studies have identified residues such as E166 to be of importance, they have missed these other residues that are distant from the substrate-binding site^40–42^. It should also be mentioned that our studies were conducted with the ancestral WA1 strain, and the currently circulating Omicron variants—all of which except for BA.3 contain a P132H mutation in 3CL^pro^—may differ in their nirmatrelvir evasion pathways. Whereas this mutation has been reported to have no direct effect on nirmatrelvir resistance, it may influence the emergence of subsequent resistance-conferring mutations^43^. It will require extensive virological, biochemical and structural studies to delineate which mutations confer resistance and how, as well as to understand how certain mutations play compensatory roles. A better understanding of the mechanisms of nirmatrelvir resistance could provide insight into the development of the next generation of 3CL^pro^ inhibitors.

At the time of writing, nirmatrelvir has been used to treat COVID-19 for only 6 months or less in most countries. SARS-CoV-2 resistance to this drug in patients has yet to be reported, and we see no appreciable difference in frequencies of the 3CL^pro^ mutations that we have uncovered in periods before and after emergency use authorization (Extended Data Fig. 8). Perhaps the absence of nirmatrelvir resistance in patients to date is due to the high drug concentrations achieved with the prescribed regimen, making it difficult for the virus to accumulate mutations in a stepwise manner. In addition, the drug is administered while the immune system is also actively eliminating the virus, including any resistant forms that may have emerged. Therefore, it makes sense to focus our surveillance effort on immunocompromised individuals on nirmatrelvir treatment for the appearance of drug-resistant virus. Past experience with other viral infections tells us that if drug resistance can be selected in vitro, it surely will occur also in vivo. Although current COVID-19 therapies have been largely administered as monotherapies, it is possible that future treatment will benefit from the use of a combination of drugs to minimize the likelihood of SARS-CoV-2 escape.

## Methods

### Biosafety

All SARS-CoV-2 passaging, infection and recombinant virus production was conducted in BSL-3 laboratories at Columbia University Irving Medical Center under procedures and guidelines approved by the Columbia University Institutional Biosafety Committee.

### Compounds

Nirmatrelvir was purchased from Aobius, ensitrelvir from Glixx Laboratories and remdesivir from Selleckchem.

### Cells

Vero E6 cells were obtained from ATCC (no. CRL-1586), HEK293T cells from ATCC (no. CRL-3216) and Vero E6-TMPRSS2-T2A-ACE2 cells from BEI Resources (no. NR-54970). Huh7-ACE2 cells were generated previously^33,44^. Cell morphology was visually confirmed before use and all cell lines tested mycoplasma negative. All cells were maintained at 37 °C under 5% CO_2_.

### In vitro selection for SARS-CoV-2 resistance to nirmatrelvir in Vero E6 cells

To select for the development of drug resistance against nirmatrelvir, WA1 (SARS-CoV-2, USA-WA1/2020 strain) was cultured in the presence of increasing concentrations of nirmatrelvir and passaged 30 times. Virus isolates recovered from culture at various passages were then characterized for their resistance to nirmatrelvir and their replication capacity.

To initiate passaging, Vero E6 cells were seeded in a 24-well plate at a density of 1 × 10^5^ cells per well in complete medium (DMEM + 10% fetal calf serum + penicillin/streptomycin), and both drug and virus were then added the following day. The drug was prepared in a threefold dilution series based on its original IC_50_. The virus was added at 5,000 50% tissue culture infectious dose (TCID_50_) per well. Three days post infection, each well was scored for cytopathic effects (CPE) in a range of 0 to 4+ based on comparison with control wells as previously described^45^, and 100 µl of the supernatant from the well with a CPE score equal to or greater than 2+ was passaged to each well in the next culture plate. The passage culture was set up in triplicate (lineages A, B and C) and passaging was performed independently—that is, viruses in lineage A were kept within the lineage A series of wells at every passage. Along with the cultures passaged with nirmatrelvir, WA1 was passaged without nirmatrelvir in two independent wells to serve as a passage control.

Values of IC_50_ for each lineage in passaging were determined based on CPE scores at day 3 of each passage; these values were derived using DeltaGraph (Red Rock Software).

### Sequencing of SARS-CoV-2 passaged in Vero E6 cells

For SARS-CoV-2 passaged in Vero E6 cells, passages were sequenced by either Sanger or Nanopore sequencing. For Sanger sequencing, viral RNA was isolated from culture supernatant with the QIAamp Viral RNA Mini Kit (Qiagen), reverse transcribed to complementary DNA with Superscript IV Reverse Transcriptase (ThermoFisher) and the priming primer (nsp5.R1) and subjected to nested PCR with Platinum SuperFi II (ThermoFisher) to obtain the full-length nsp5 gene. Primers for the first PCR were nsp5.F1: 5′-GTAGTGATGTGCTATTACCTCTTACGC-3′ and nsp5.R1: 5′-GCAAAAGCAGACATAGCAATAATACC-3′. Primers for the second PCR were nsp5.F2: 5′-CTTCAGTAACTCAGGTTCTGATGTTCT-3′ and nsp5.R2: 5′-ACCATTGAGTACTCTGGACTAAAACTAAA-3′. Both PCRs were run under the same conditions: 98 °C for 30 s, 25 cycles of 98 °C for 15 s, 60 °C for 10 s and 72 °C for 1 min, followed by 72 °C for 5 min. PCR products were purified and sequenced (Genewiz). Mixtures of viruses were determined by inspection of sequencing chromatograms. Sequences were analysed using Lasergene software (DNASTAR).

For Nanopore sequencing, viral RNA was isolated from culture supernatant with the QIAamp Viral RNA Mini Kit (Qiagen), Midnight RT PCR Expansion and Rapid Barcoding kits (Oxford Nanopore) were used for amplification and barcode-overlapping 1,200-base pair (bp) amplicons were tiled across the viral genome^46,47^. An Oxford Nanopore GridION with R9.4.1 flow cells was used for sequencing. Basecalling was performed in MinKNOW v.22.05.1. Consensus sequence generation was performed using the ONT Epi2Me ARTIC Nextflow pipeline v.0.3.16 (https://github.com/epi2me-labs/wf-artic). Pangolin 4.0.6 with UShER v.1.6 was used for parsimony-based lineage assignment. Sequences have been deposited at GenBank (nos. ON924329-ON924335 and ON930401-ON930431) (Supplementary Table 3).

### Inhibition assay with SARS-CoV-2 passaged in Vero E6 cells

To characterize the inhibition of passaged viruses, each virus was first propagated in Vero E6 cells in the absence of drug and titrated using the Reed–Muench method^48^. Vero E6 cells were then seeded in 96-well plates at a density of 1.5 × 10^4^ cells per well in complete medium. The following day, the virus was inoculated at a dose of 500 TCID_50_ per well and a twofold dilution series of inhibitor added in quadruplicate. Three days post infection, the level of CPE was scored and IC_50_ was derived by fitting a nonlinear regression curve to the data in GraphPad Prism v.9.4 (Dotmatics).

### Growth assay with SARS-CoV-2 passaged in Vero E6 cells

The fitness of passaged viruses was characterized by viral growth assay. Vero E6 cells were seeded in 96-well plates at a density of 1.5 × 10^4^ cells per well in complete medium. The following day, the virus was inoculated at a dose of 200 TCID_50_ per well in quadruplicate. At 6 h post infection, free virions in the culture were removed by changing of the medium twice. At 11, 24, 35 and 49 h post infection, 50 µl of culture supernatant from each well was collected and replenished with an equivalent volume of fresh medium. Viral RNA from each time point was purified using a PureLink Pro 96 Viral RNA/DNA Purification Kit (ThermoFisher), and viral copy number in each sample was then estimated by quantitative PCR with reverse transcription using TaqPath 1-Step RT–qPCR Master Mix (ThermoFisher) and a 2019-nCov CDC EUA Kit (Integrated DNA Technologies) with a 7500 Fast Dx Real-Time PCR Instrument (Applied Biosystems).

### In vitro selection for SARS-CoV-2 resistance to nirmatrelvir in Huh7-ACE2 cells

To conduct selection at scale to observe as many resistance pathways as possible, SARS-CoV-2 infection was conducted in five 96-well plates thereby facilitating 480 independent selection lineages. We hypothesized that the use of limited number of cells would allow for a ‘bottleneck effect’ to occur, which would enable observation of rarer events that may be outcompeted from a larger population.

To initiate passaging, 3 × 10^4^ Huh7-ACE2 cells per well were seeded in complete medium in five 96-well plates. The following day, all wells were infected with 0.05 MOI of SARS-CoV-2-mNeonGreen (a fluorescent reporter variant of USA-WA1/2020, gift of P.-Y. Shi)^27^ without drug to generate passage 0. For each successive passage, cells were seeded the day before infection and the drug and virus then added 3–4 days after infection of the previous passage. The drug was initially added at 25 nM and then doubled every other successive passage. Viruses were transferred between passages by overlaying 50 µl of the supernatant from the previous passage. After 16 passages all 54 wells positive for mNeonGreen signal were sequenced, from which 53 lineages could be determined.

### Inhibition assay with SARS-CoV-2 passaged in Huh7-ACE2 cells

To characterize the inhibition of passaged viruses, each of the viruses were first propagated in Huh7-ACE2 cells in the absence of drug and titrated using the Reed–Muench method^48^. Huh7-ACE2 cells were then seeded in 96-well plates at a density of 2 × 10^4^ cells per well in complete medium. The following day, the virus was inoculated at a dose of 0.05 MOI per well and a fivefold dilution series of inhibitor added in triplicate. At 24 h post infection, supernatant was aspirated and cells were fixed with 4% paraformaldehyde in PBS and stained with DAPI. Cells were then imaged for DAPI and green fluorescent protein using IN Cell 2000 (GE) and analysed with CellProfiler v.4.0.7 (ref. ^49^). The IC_50_ level was then derived by fitting a nonlinear regression curve to the data in GraphPad Prism v.9.4 (Dotmatics).

### Sequencing of SARS-CoV-2 passaged in Huh7-ACE2 cells

For SARS-CoV-2 passaged in Huh7-ACE2 cells, passages were sequenced by Illumina NGS. Viral RNA was first extracted using a PureLink Pro 96 Viral RNA/DNA Purification Kit (ThermoFisher). Reverse transcription was carried out using a Maxima H Minus First Strand cDNA Synthesis Kit (ThermoFisher) with random hexamers according to the manufacturer’s instructions. Briefly, 13.75 µl of viral RNA was mixed with 0.25 µl of random hexamers (50 ng µl^–1^) and 1 µl of deoxynucleotide triphosphates (dNTPs) (10 mM), and incubated at 65 °C for 5 min followed by 4 °C for 1 min. A mixture containing 4 µl of 5× RT buffer, 0.25 µl of enzyme mix (containing Maxima H Minus RT and RNase inhibitor) and 0.75 µl of H_2_O was added to each sample and the reactions incubated at 25 °C for 10 min, 55 °C for 30 min and 85 °C for 5 min.

Sequencing libraries were prepared by amplification of either nine fragments tiled across the 3CL^pro^ open reading frame and adjacent nsp4/5 and nsp5/6 cut sites, or nine fragments containing each of the remaining 3CL^pro^ cut sites (see Supplementary Table 4 for primer sequences). Primers amplifying nonadjacent fragments of 3CL^pro^ were pooled and reactions carried out in technical duplicate, for a total of four first-round PCRs per sample. Each first-round PCR contained the following components: 1 µl of cDNA, 0.25 µl of 100 µM pooled primers, 0.4 µl of 10 mM dNTPs, 2 µl of 10× Taq buffer, 0.1 µl of Taq DNA polymerase (Enzymatics) and 16.25 µl of H_2_O. Cycling conditions were as follows: (1) 94 °C for 3 min, (2) 94 °C for 30 s, (3) 54 °C for 20 s, (4) 72 °C for 30 s, (5) return to step 2 for 34 additional cycles, (6) 72 °C for 3 min and (7) hold at 4 °C.

Products from the four first-round PCRs for each sample were pooled and gel purified, and second-round indexing PCR was carried out for each sample with the following reagents: 1 µl of template DNA, 0.25 µl of each 100 µM indexing primer, 0.4 µl of 10 mM dNTPs, 2 µl of 10× Taq buffer, 0.1 µl of Taq DNA polymerase and 16.25 µl of H_2_O. Cycling conditions were as follows: (1) 94 °C for 3 min, (2) 94 °C for 30 s, (3) 54 °C for 20 s, (4) 72 °C for 30 s, (5) return to step 2 for six additional cycles, (6) 72 °C for 3 min and (7) hold at 4 °C.

Second-round PCR products were pooled, gel purified and sequenced on an Illumina NextSeq system with 150-bp single-end reads. For select samples, sequences were confirmed using nanopore sequencing (Plasmidsaurus). For samples P16-2D9, P12-1A4 and 4-3A1, the original Illumina sequencing results were replaced by nanopore sequencing results.

For each sample, mutations and their frequencies were identified using the V-pipe computational pipeline (v.2.99.2)^50^, with Wuhan-Hu-1 (GenBank accession no. MN908947) set as the reference sequence. Frequency thresholds for reporting mutations were set at 5 and 10% for Illumina and nanopore sequencing, respectively. Supplementary Table 1 shows the absolute frequencies of mutations within each sample. Raw sequencing data have been deposited with NCBI Short Read Archive under BioProject Accession ID PRJNA852265 (Supplementary Table 5 gives SRA Accession IDs for each sample). These sequences were clustered for Fig. 2c using ‘seaborn.clustermap’ under default settings, which utilizes the UPGMA algorithm through SciPy^51,52^. The phylogenetic analysis shown in Fig. 3a was produced in Geneious Prime v.2022.1 with PHYML extension, using the GTR substitution model with the optimization conditions of topology/length/rate.

### Pathway analysis for SARS-CoV-2 passaged in Huh7-ACE2 cells

Figure 3b was constructed from lineages containing only those mutations found most commonly in passage 16: T21I, T304I, A173V, E166V, P252L, S144A and L50F. These lineages were determined based on the frequencies of the corresponding mutations in a given well at each passage. Pairs of mutants whose frequencies summed to greater than 100% were assumed to co-occur on the same allele. The same logic was extended to identify triple and quadruple mutants, such that if each pairwise sum of frequencies within a group of mutations was greater than 100%, all mutations within that group were assumed to occur together. The order in which mutations in a given lineage arose was imputed either from stepwise appearance over time (for example, passage 4 has mutation 1 and passage 8 has mutations 1 and 2 at a total combined frequency greater than 100%) with increasing frequencies, or, in cases in which two mutations arose between sequenced passages and were deemed to co-occur in a single virus, by their relative frequencies (for example, if passage 4 has no mutations and passage 8 has mutation 1 at 99% frequency and mutation 2 at 30% frequency, mutation 1 was assumed to have arisen first). See Supplementary Table 2 for the datapoints used in this analysis.

### Recombinant SARS-CoV-2 production

A reverse genetics system based on the pBeloBAC11 bacterial artificial chromosome (BAC), containing the SARS-CoV-2 genome with a NanoLuc luciferase reporter replacing ORF7a^53^ (gift of L.-M. Sobrido), was used to produce recombinant SARS-CoV-2 harbouring 3CL^pro^ mutations. Mutant BACs were produced as previously described^33^; see Supplementary Table 6 for a list of mutagenic primers used. These BACs (2 µg each) were then transfected into HEK293T cells in 12-well plates in triplicate using Lipofectamine 3000 Transfection Reagent (ThermoFisher) according to the manufacturer’s instructions. Two days post transfection, cells were pooled and overlaid onto Vero E6-TMPRSS2-T2A-ACE2 cells in 25 cm^2^ flasks. After 3 days, supernatant was collected from these cells, clarified by centrifugation then used to infect Vero E6 cells in 75 cm^2^ flasks. Four days post infection, supernatant was harvested, clarified by centrifugation and aliquoted. Viruses were stored at −80 °C before use. An aliquot of all recombinant viruses was confirmed by nanopore sequencing for the mutation of interest, and for purity before use.

### Inhibition assay with recombinant SARS-CoV-2

Viruses were first titrated to normalize input. To characterize inhibition, Huh7-ACE2 cells were seeded at a density of 2 × 10^4^ cells per well in 96-well plates. The following day, cells were infected with 0.05 MOI of virus and treated with inhibitor in a fivefold dilution series. One day post infection, cells were lysed and luminescence quantified using the Nano-Glo Luciferase Assay System (Promega), according to the manufacturer’s instructions, with a SpectraMax i3× Multi-Mode Microplate Reader (Molecular Devices) using SoftMax Pro v.7.0.2 software (Molecular Devices). IC_50_ values were derived by fitting a nonlinear regression curve to the data in GraphPad Prism v.9.4 (Dotmatics).

### Growth assay with recombinant SARS-CoV-2

Viruses were first titrated to normalize input. To characterize fitness, Huh7-ACE2 cells were seeded at a density of 2 × 10^4^ cells per well in 96-well plates. The following day, cells were infected with 0.01 MOI of virus. At 12, 24, 36 and 48 h post infection, cells were lysed and luminescence quantified using the Nano-Glo Luciferase Assay System, according to the manufacturer’s instructions, with a SpectraMax i3× Multi-Mode Microplate Reader using SoftMax Pro v.7.0.2 software.

### Retrieval of clinical mutation frequencies

COVID-19 CG was used to retrieve all clinically observed 3CL^pro^ mutations from the Global Initiative on Sharing Avian Influenza Data on 26 June 2022, either since the start of the COVID-19 pandemic or for the periods 26 March–26 June 2022 and 22 September–22 December 2021 (refs. ^31,54^).

### Materials availability

Materials used in this study will be made available under an appropriate materials transfer agreement.

### Reporting summary

Further information on research design is available in the Nature Portfolio Reporting Summary linked to this article.

## Online content

Any methods, additional references, Nature Portfolio reporting summaries, source data, extended data, supplementary information, acknowledgements, peer review information; details of author contributions and competing interests; and statements of data and code availability are available at 10.1038/s41586-022-05514-2.

## Supplementary information

Reporting Summary

Supplementary Table 1 Raw sequencing results for passaging in Huh7-ACE2 cells. Samples are denoted as passage number, followed by well number. Cut site mutations are denoted as ‘cs’.

Supplementary Table 2 Passage transitions used for construction of the pathway analysis in Fig. 3b.

Supplementary Table 3 GenBank Accession IDs for sequences from passaging in Vero E6 cells.

Supplementary Table 4 Oligos used for NGS.

Supplementary Table 5 SRA Accession IDs for raw sequencing data from passaging in Huh7-ACE2 cells.

Supplementary Table 6 Oligos used for site-directed mutagenesis to produce isogenic recombinant SARS-CoV-2.

## Data Availability

All experimental data are provided in the manuscript. The sequences of mutants from passaging in Vero E6 cells have been deposited at GenBank (nos. ON924329–ON924335 and ON930401–ON930431). The raw NGS data of passaging in Huh7-ACE2 cells are available from the NCBI Sequence Read Archive under BioProject Accession ID PRJNA852265. The structures of the 3CL^pro^–nirmatrelvir and 3CL^pro^–ensitrelvir complexes were downloaded from PDB under accession codes 7VH8 and 7VU6, respectively. The Wuhan-Hu-1 sequence used for alignment was downloaded from GenBank (accession no. MN908947).

## Code availability

Sequencing data processing and visualization was performed using the ONT ARTIC Nextflow pipeline (https://github.com/epi2me-labs/wf-artic) and the V-pipe computational pipeline (https://github.com/cbg-ethz/V-pipe) and clustering was performed using seaborn (https://github.com/mwaskom/seaborn), which utilizes SciPy (https://github.com/scipy/scipy), all of which are publicly available software packages.
