# Supplementary material for: Multiple pathways for SARS-CoV-2 resistance to nirmatrelvir
Source: Nature. 2022 Nov 9;613(7944):558–64. doi: 10.1038/s41586-022-05514-2 (PMC9849135; doi:10.1038/s41586-022-05514-2)
Supplement: Supplementary file 1 — Reporting Summary [file 41586_2022_5514_MOESM1_ESM.pdf]

nature portfolio

Corresponding author(s): Alejandro Chavez, David D. Ho

Last updated by author(s): Oct 17, 2022

Reporting Summary

Nature Portfolio wishes to improve the reproducibility of the work that we publish. This form provides structure for consistency and transparency in reporting. For further information on Nature Portfolio policies, see our Editorial Policies and the Editorial Policy Checklist.

Statistics

For all statistical analyses, confirm that the following items are present in the figure legend, table legend, main text, or Methods section.

n/a	Confirmed	
☐	☒	The exact sample size (*n*) for each experimental group/condition, given as a discrete number and unit of measurement
☐	☒	A statement on whether measurements were taken from distinct samples or whether the same sample was measured repeatedly
☐	☒	The statistical test(s) used AND whether they are one- or two-sided<br>*Only common tests should be described solely by name; describe more complex techniques in the Methods section.*
☒	☐	A description of all covariates tested
☐	☒	A description of any assumptions or corrections, such as tests of normality and adjustment for multiple comparisons
☐	☒	A full description of the statistical parameters including central tendency (e.g. means) or other basic estimates (e.g. regression coefficient) AND variation (e.g. standard deviation) or associated estimates of uncertainty (e.g. confidence intervals)
☐	☒	For null hypothesis testing, the test statistic (e.g. *F*, *t*, *r*) with confidence intervals, effect sizes, degrees of freedom and *P* value noted<br>*Give P values as exact values whenever suitable.*
☒	☐	For Bayesian analysis, information on the choice of priors and Markov chain Monte Carlo settings
☒	☐	For hierarchical and complex designs, identification of the appropriate level for tests and full reporting of outcomes
☒	☐	Estimates of effect sizes (e.g. Cohen's *d*, Pearson's *r*), indicating how they were calculated

Our web collection on statistics for biologists contains articles on many of the points above.

Software and code

Policy information about availability of computer code

Data collection	SoftMax Pro 7.0.2 (Molecular Devices, LLC) was used to measure luminescence in the inhibition assays.
Data analysis	DeltaGraph version 7 (Red Rock Software) and GraphPad Prism version 9.4 (Dotmatics) were used for determination of IC50 values and statistical tests. GraphPad version 9.4 was used for data visualization. Lasergene software version 17 (DNASTAR) was used for Sanger sequencing analysis. MinKNOW v22.05.1, ONT Epi2Me ARTIC Nextflow pipeline v0.3.16, Pangolin 4.0.6 with UShER v1.6, and V-pipe version 2.99.2 was used for next-generation sequencing analysis. seaborn v0.10.0, which utilizes SciPy, was used for clustering. Geneious Prime v2022.1 with PHYML extension was used for phylogenetic analyses. COVID-19 CG was used for querying sequences deposited to GISAID. CellProfiler version 4.0.7 was used for image analysis.

For manuscripts utilizing custom algorithms or software that are central to the research but not yet described in published literature, software must be made available to editors and reviewers. We strongly encourage code deposition in a community repository (e.g. GitHub). See the Nature Portfolio guidelines for submitting code & software for further information.

Data

Policy information about availability of data

All manuscripts must include a data availability statement. This statement should provide the following information, where applicable:

- Accession codes, unique identifiers, or web links for publicly available datasets
- A description of any restrictions on data availability
- For clinical datasets or third party data, please ensure that the statement adheres to our policy

All experimental data are provided in the manuscript. The sequences of mutants from passaging in Vero E6 cells have been deposited to GenBank (ON924329-ON924335, ON930401-ON930431). The raw next-generation sequencing data of passaging in Huh7-ACE2 cells are available from the NCBI Short Read Archive under BioProject Accession ID PRJNA852265. The structures of the 3CLpro-nirmatrelvir complex and 3CLpro-ensitrelvir complex were downloaded from PDB under accession codes 7VH8 and 7VU6, respectively. The Wuhan-Hu-1 sequence used for alignment was downloaded from GenBank (accession no. MN908947).

Human research participants

Policy information about studies involving human research participants and Sex and Gender in Research.

Reporting on sex and gender	N/A, this study did not involve human research participants.
Population characteristics	N/A, this study did not involve human research participants.
Recruitment	N/A, this study did not involve human research participants.
Ethics oversight	N/A, this study did not involve human research participants.

Note that full information on the approval of the study protocol must also be provided in the manuscript.

Field-specific reporting

Please select the one below that is the best fit for your research. If you are not sure, read the appropriate sections before making your selection.

☒ Life sciences ☐ Behavioural & social sciences ☐ Ecological, evolutionary & environmental sciences

For a reference copy of the document with all sections, see nature.com/documents/nr-reporting-summary-flat.pdf

Life sciences study design

All studies must disclose on these points even when the disclosure is negative.

Sample size	No sample size calculation was performed. We utilized sample sizes as used in similar studies which allowed for results which could be replicated (e.g., Iketani et al 2022 Nature, Liu et al 2022 Nature, Wang et al 2021 Nature).
Data exclusions	No data were excluded.
Replication	The inhibition assay in Fig. 2b and the growth assay in Fig. 3b were repeated independently twice. The inhibition assay in Fig. 4a were repeated independently three times.
Randomization	As this is an observational study, randomization is not relevant.
Blinding	As this is an observational study, investigators were not blinded.

Reporting for specific materials, systems and methods

We require information from authors about some types of materials, experimental systems and methods used in many studies. Here, indicate whether each material, system or method listed is relevant to your study. If you are not sure if a list item applies to your research, read the appropriate section before selecting a response.

Materials & experimental systems

n/a	Involved in the study
☒	☐ Antibodies
☐	☒ Eukaryotic cell lines
☒	☐ Palaeontology and archaeology
☒	☐ Animals and other organisms
☒	☐ Clinical data
☐	☒ Dual use research of concern

Methods

n/a	Involved in the study
☒	☐ ChIP-seq
☒	☐ Flow cytometry
☒	☐ MRI-based neuroimaging

Eukaryotic cell lines

Policy information about cell lines and Sex and Gender in Research

Cell line source(s)	Vero E6 cells were obtained from ATCC (Catalog #CRL-1586), HEK293T cells were obtained from ATCC (Catalog #CRL-3216), and Vero E6-TMPRSS2-T2A-ACE2 cells were obtained from BEI Resources (Catalog #NR-54970). Huh7-ACE2 cells were generated previously (refs 33,40).
Authentication	Cell lines were purchased from authenticated vendors, and morphology was also confirmed visually prior to use.
Mycoplasma contamination	Cell lines tested mycoplasma negative.
Commonly misidentified lines (See ICLAC register)	No commonly misidentified cell lines were used in this study.

Dual use research of concern

Policy information about dual use research of concern

Hazards

Could the accidental, deliberate or reckless misuse of agents or technologies generated in the work, or the application of information presented in the manuscript, pose a threat to:

No	Yes
☒	☐ Public health
☒	☐ National security
☒	☐ Crops and/or livestock
☒	☐ Ecosystems
☒	☐ Any other significant area

Experiments of concern

Does the work involve any of these experiments of concern:

No	Yes
☒	☐ Demonstrate how to render a vaccine ineffective
☐	☒ Confer resistance to therapeutically useful antibiotics or antiviral agents
☒	☐ Enhance the virulence of a pathogen or render a nonpathogen virulent
☒	☐ Increase transmissibility of a pathogen
☒	☐ Alter the host range of a pathogen
☒	☐ Enable evasion of diagnostic/detection modalities
☒	☐ Enable the weaponization of a biological agent or toxin
☒	☐ Any other potentially harmful combination of experiments and agents

Precautions and benefits

Biosecurity precautions	All experiments were conducted in a Biosafety Level 3 (BSL-3) facility.
Biosecurity oversight	Prior to conducting this work, the protocol was reviewed and approved by Columbia University's Institutional Biosafety Committee (IBC).
Benefits	Understanding of the mutations that confer nirmatrelvir resistance, as well as the mechanisms by which SARS-CoV-2 acquires such resistance, is critical for clinical surveillance of nirmatrelvir resistance and for the development of future protease inhibitors.

Communication benefits	Communication of these results will allow for clinical surveillance and appropriate use of nirmatrelvir, as well as provide insight into development of the next generation of protease inhibitors. Some of these mutant viruses have already been described elsewhere. Furthermore, as these described viruses remain susceptible to other therapeutic agents and arose naturally, we believe that communication of our data outweigh the risks.
